# Impact of the Surviving Sepsis Campaign implementation on severe sepsis outcome

**DOI:** 10.1186/cc10162

**Published:** 2011-06-22

**Authors:** E Silva

**Affiliations:** 1 Hospital Israelita Albert Einstein, São Paulo - SP, Brazil

## Introduction

Sepsis is associated with high morbi-mortality rates and evidence-based strategy implementation could improve outcome.

## Objective

To evaluate the impact of the 6-hour Surviving Sepsis Campaign (SSC) bundle on mortality in a tertiary hospital.

## Methods

A multifaceted intervention to facilitate compliance with selected guideline recommendations in the intensive care unit, emergency department, and wards in our hospital was implemented. Data were collected in two periods, before and after implementation of the protocol, from July 2005 to December 2008. The first period was called the Control Group from July 2005 to March 2006 and the Protocol Group from April 2006 to December 2008. SSC was implemented in April 2006. Compliance to the 6-hour SSC bundle was measured in both periods, as well as outcome.

## Results

A total of 414 patients were enrolled, 92 in the Control Group and 322 in the Protocol Group. Mean age was 66 ± 19 years, mean APACHE II score was 24.1 ± 7.5, and 42% were female. Hospital LOS in the Control Group was 37 ± 44 days and in the Protocol Group was 47 ± 90 (*P *= 0.36). ICU LOS were similar, 14 ± 17 days and 14 ± 35 days, respectively. The 6-hour bundle adherence has significantly increased from 10% in the Control Group to 28% in the Protocol Group (*P *= 0.001). Differences between 6-hour bundle variables are shown in Table 1. The mortality rate decreased after protocol implementation from 57% to 38% (*P *= 0.001).

**Table 1:** Compliance to each SSC 6-hour bundle variable

Variable (%)	Overall (*n *= 414)	Control (*n *= 92)	Protocol (*n *= 322)	*P *value
Blood culture	69	55	73	0.001
Lactate measurement	83	70	87	<0.0005
Central venous oxygen saturation	62	55	65	0.068
Central venous pressure	65	48	70	<0.0005
Early large spectrum antibiotics	89	86	89	0.46
All variables	24	10	28	0.001

## Conclusion

Implementation of the SSC 6-hour bundle was associated with lower mortality.

